# Exploring With Transcriptomic Approaches the Underlying Mechanisms of an Essential Oil-Based Phytogenic in the Small Intestine and Liver of Pigs

**DOI:** 10.3389/fvets.2021.650732

**Published:** 2021-08-11

**Authors:** Jeremy Le Coz, Suzana Ilic, Silvia Fibi-Smetana, Gerd Schatzmayr, Mathias Zaunschirm, Bertrand Grenier

**Affiliations:** ^1^BIOMIN Research Center, BIOMIN Holding GmbH, Tulln, Austria; ^2^BIOMIN Holding GmbH, Getzersdorf, Austria

**Keywords:** swine, RNA-Seq, essential oil, inflammation, oxidative stress, gene expression, antibiotic growth promoter

## Abstract

Phytogenics are plant-based feed additives utilized in animal nutrition to support animal growth and health. Worldwide restrictions and bans on the use of antibiotic growth promoters resulted in an increased demand for in-feed alternatives including phytogenics. However, several challenges remain for technology readiness in animal industry, especially regarding the standardization of the ingredients as well as our knowledge on the cellular mechanisms underlying their biological effects. In the present study, 32 weaned piglets were allocated for 28 days to four experimental diets, a control diet, a phytogenic feed additive (PFA) diet, or the same two diets but with the addition of oxidized oil (OO) at 10%. The last two diets aimed at evaluating the antioxidant properties of PFA. At the end of the trial, the ileum and the liver of the pigs were sampled, and RNA were isolated for profiling their transcriptome *via* RNA sequencing (RNA-Seq). In the ileum, the gene set enrichment analysis showed that the activity of several immune pathways (NF-kB, interferon α/β, antimicrobial peptide, and collagen pathways) was reduced in piglets fed PFA compared to the control piglets. As expected, the addition of OO induced strong effects on the liver transcriptome and most likely accounted for the significant growth impairment. The likelihood ratio test across the four diets revealed a global response driven by the oxidative stress challenge with hundreds of genes associated with fatty acid β-oxidation and peroxisome in the liver. The expression levels of those genes in the piglets fed OO+PFA were much less affected by the challenge. Collectively, the effects seen at day 28 suggest that substances in the PFA formulation provide anti-inflammatory and antioxidant properties. The use of RNA-Seq in animal nutrition allows exploring and deciphering novel mechanisms of natural growth promoters.

## Introduction

Young piglets are more susceptible to stressors than older pigs, including bacterial infections, intestinal inflammatory disorders, or oxidative stress. To maintain growth efficiency and animal health, antibiotic growth promoters (AGPs) have widely been used in the pig industry, especially in nurseries (1). However, the concern over antibiotic resistance and general consumer pressure have led to restrictions on the excessive use of AGPs, especially in the European Union with a ban on AGPs since 2006 (2–5). The past 10 years have seen the constant raise and demand for feed additives promoting growth and enhancing general health as alternatives to AGPs. These include probiotics, organic acids, phytogenics, prebiotics, enzymes, or antimicrobial peptides (6–8). Some have been successful in bridging the gap between technology concept and technology readiness for animal industry. This includes standardization of the ingredients, optimization of the formulation for minimal inclusion rate, or implementation of new feed technology such as encapsulation [e.g., for phytogenics (9)]. However, several challenges remain with these alternatives for ensuring the long-term sustainability of pig production (10, 11). There is a lack of information on their mechanism of action; some of these products lack consistency, and their efficacies may vary among farms and locations. To mitigate these challenges, manufacturers should provide analytical methods and robust published research to back their claims.

Feed phytogenics, also referred to as botanicals or phytochemicals, are plant-based feed additives utilized in animal nutrition. They are derived from herbs, spices, and extracts such as essential oils. Essential oils have been widely used over the centuries as traditional medicines to improve health or cure diseases in humans (12, 13). The presence of functional hydroxyl or alkyl groups from the phenolic compounds (e.g., carvacrol, thymol, and linalool) in essential oils is known to be responsible for their antimicrobial, anti-inflammatory, and antioxidant properties. The number of scientific reviews and studies on the use of essential oil and aromatic plants as feed additives in animal nutrition has exploded in the past 5 years. However, there is a thin line between a successful formula and a mere mixture of different components. The mechanism of action of phytochemicals is not clearly understood but may depend on the composition of the active ingredients in the product being used. In addition, it is difficult to ascertain in a blend of ingredients which exact component is responsible for which effect. To study and conclude on the mode of action of such feed additives, it is therefore essential to use standardized formulation, consistent sourcing, and manufacturing methods. The use of high-throughput technologies such as next-generation sequencing has now become a popular tool to understand the cellular mechanisms underlying the effects of biological compounds, such as the mode of action of phytogenics.

In the present study, RNA sequencing (RNA-Seq) was employed for investigating the transcriptome of piglets fed an essential oil-based phytogenic for 28 days. The effects on the global gene expression were first determined in the small intestine of the piglets in normal conditions in order to provide new data regarding the interaction of this phytogenic feed additive (PFA) with the intestinal host. Secondly, within the same experiment, an oxidative stress challenge was implemented *via* addition of oxidized oil (14) in order to evaluate the antioxidant capacity of the PFA. The efficacy of PFA to neutralize the effects was evaluated in the liver of the piglets by a second RNA-Seq analysis.

## Results

### Effect of PFA on Gene Patterns and Functions in the Ileum at Day 28

**Figure 1:**
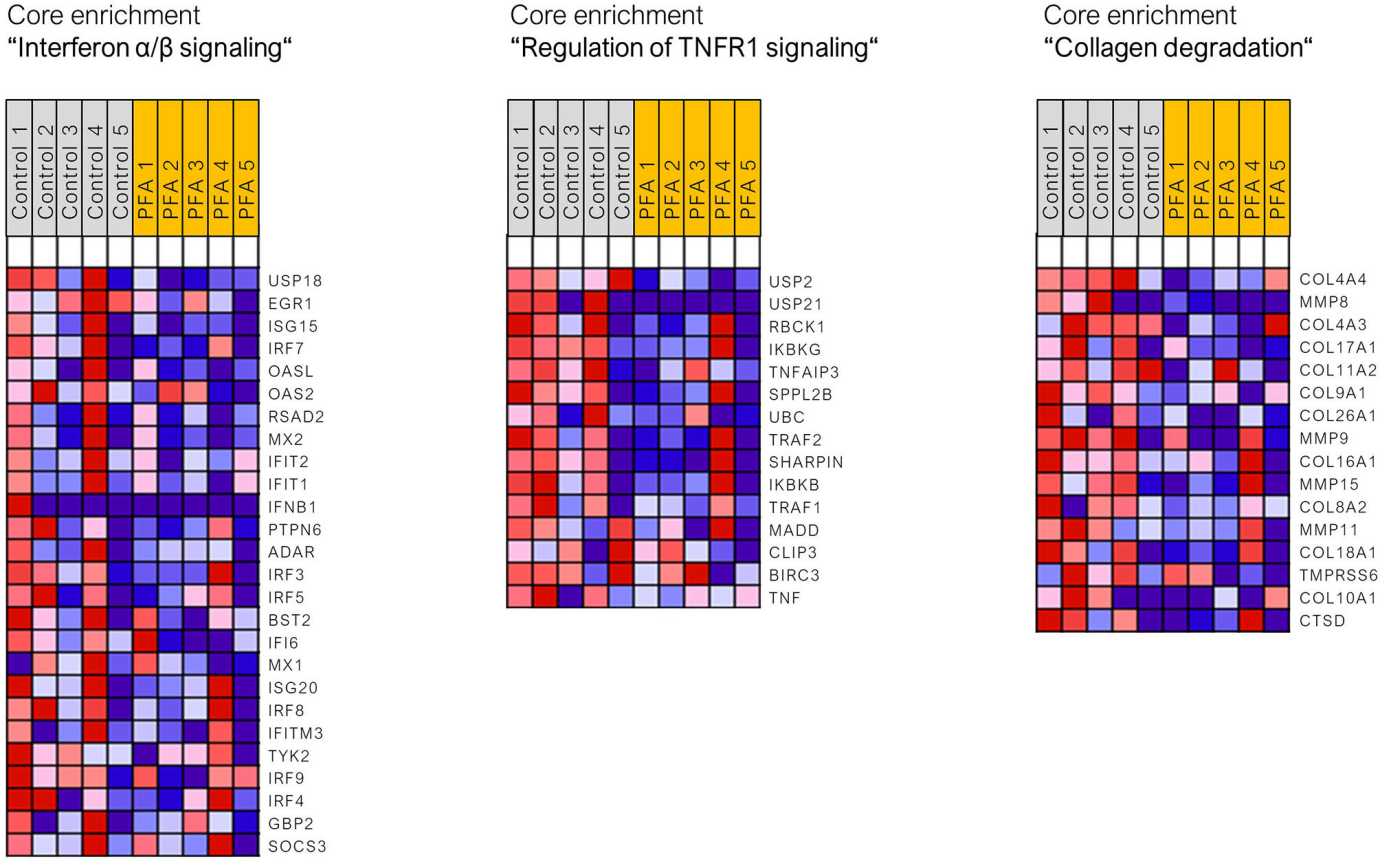
Heat map of the genes found in the core enrichment for the respective signaling pathways. Expression values are represented as colors, where the range of colors (red, pink, light blue, and dark blue) shows the range of expression values (high, moderate, low, and lowest).

Read mapping with STAR aligner into the *Sus scrofa* genome assembly Sscrofa11.1 was high, ranging from 85.1 to 91.6% (with five piglets per group only from unchallenged groups; two groups; 10 samples). Using the standard cutoff values for RNA-Seq analysis (FDR ≤ 0.05, fold change > |2|), only four DEGs (differentially expressed genes) were found between the control and PFA groups (*PRR15, ANPEP, UCHL5*, and *NOTUM*). It seemed that one animal for the PFA group responded differently than the others to the treatment. Analysis of data with Grubb's test substantiated this assumption for that pig, for which the critical outlier *Z*-value of 1.71 was exceeded in 1866 genes (this animal was still included in the final pathway analysis). Due to the limitations of functional analysis with a small list of DEGs, the gene set enrichment analysis (GSEA) was applied for this dataset, using the full dataset of genes. The gene sets and pathways significantly enriched in each phenotype (i.e., control or PFA; FDR ≤ 0.05) are listed in Supplementary Table 1. GSEA showed that the activity of the following gene sets, interferon α/β signaling, TNFR1-induced-NF-κB signaling pathway, RHO GTPases activate NADPH oxidases, regulation of TNFR1 signaling, antimicrobial peptides, TRAF6-mediated IRF7 activation, collagen chain trimerization, interleukin 7 signaling, or collagen degradation, was reduced in the PFA group compared to the control group (FDR *p*-value <0.05). Figure 1 shows three of those enriched pathways (with normalized enrichment scores ranging between 1.69 and 2.34) and the respective gene sets found in the core enrichment. Interestingly, pathways related to collagen assembly, degradation of the extracellular matrix, and chemokine receptor bind chemokines were also found to be overrepresented when using a list of downregulated genes by PFA issued with less strict RNA-Seq cutoff values (unadjusted *p*-value ≤ 0.05, fold change > 1.5 or < −1.5; data not shown; Reactome pathway database was used for the overrepresentation analysis).

Confirmation of sequencing results is typically done by qPCR on a few genes. Five genes (*CCL20, IRF7, TOLLIP, TRAF2*, and *GSDMD*) were selected and quantified in the same ileum samples by qPCR. The five genes were selected based on their occurrence in the enriched pathways in GSEA, as well as on their fold changes. The correlation analysis (X, Y pairs) between fold changes observed in RNA-Seq and in qPCR showed a Pearson correlation coefficient of *r* = 0.89 (*p* = 0.038).

### Effect of PFA and Oxidized Oil Challenge on Gene Patterns and Functions in the Liver at Day 28

For the 24 RNA-Seq paired-end libraries issued from the liver samples (with six piglets per group from unchallenged and challenged groups; four groups; 24 samples), reads mapping to the reference pig genome ranged from 79.3 to 90.8%, except for one animal with 65.2%. This sample, from the OO + PFA group, was excluded from the RNA-Seq analysis due to suspicion of RNA degradation observed during read quality control (C bias in the base distribution, heavy polyA tail selection, and the lowest percentage of mapped reads).

**Figure 2:**
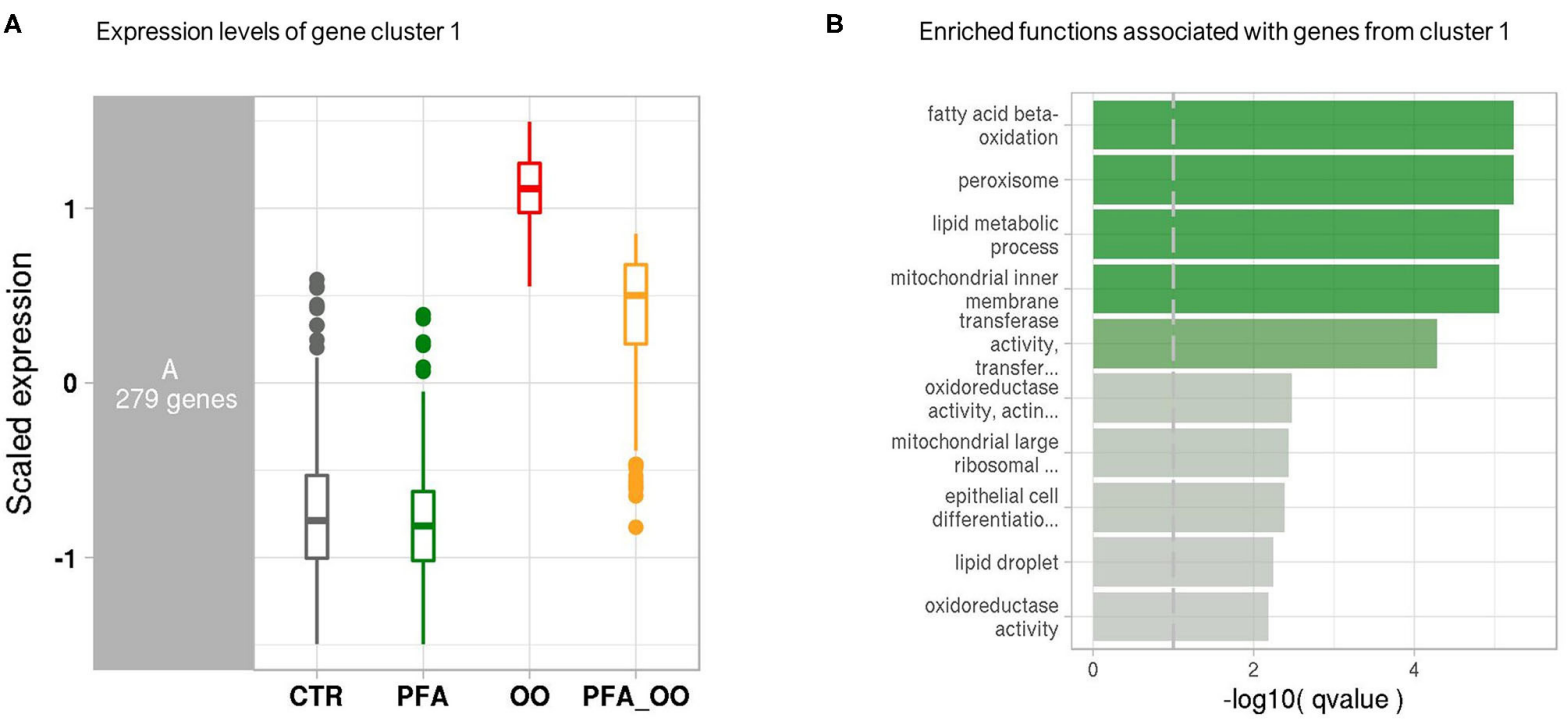
Co-expression network modules and functions. **(A)** Boxplot of expression levels per condition of the gene cluster 1, exhibiting similar expression pattern. Control (CTR), Phytogenic Feed Additive (PFA), Oxidized Oil (OO), and Oxidized Oil + Phytogenic Feed Additive (PFA_OO). Expression values (*y*-axis) are centered to the mean and scaled to the standard deviation for each gene. **(B)** Bar plot generated from the overrepresentation analysis and showing the top enriched functions associated with the genes from cluster 1. Bars are colored according to the negative logarithm of the hypergeometric test *q*-value (threshold set at 0.1).

**Figure 3:**
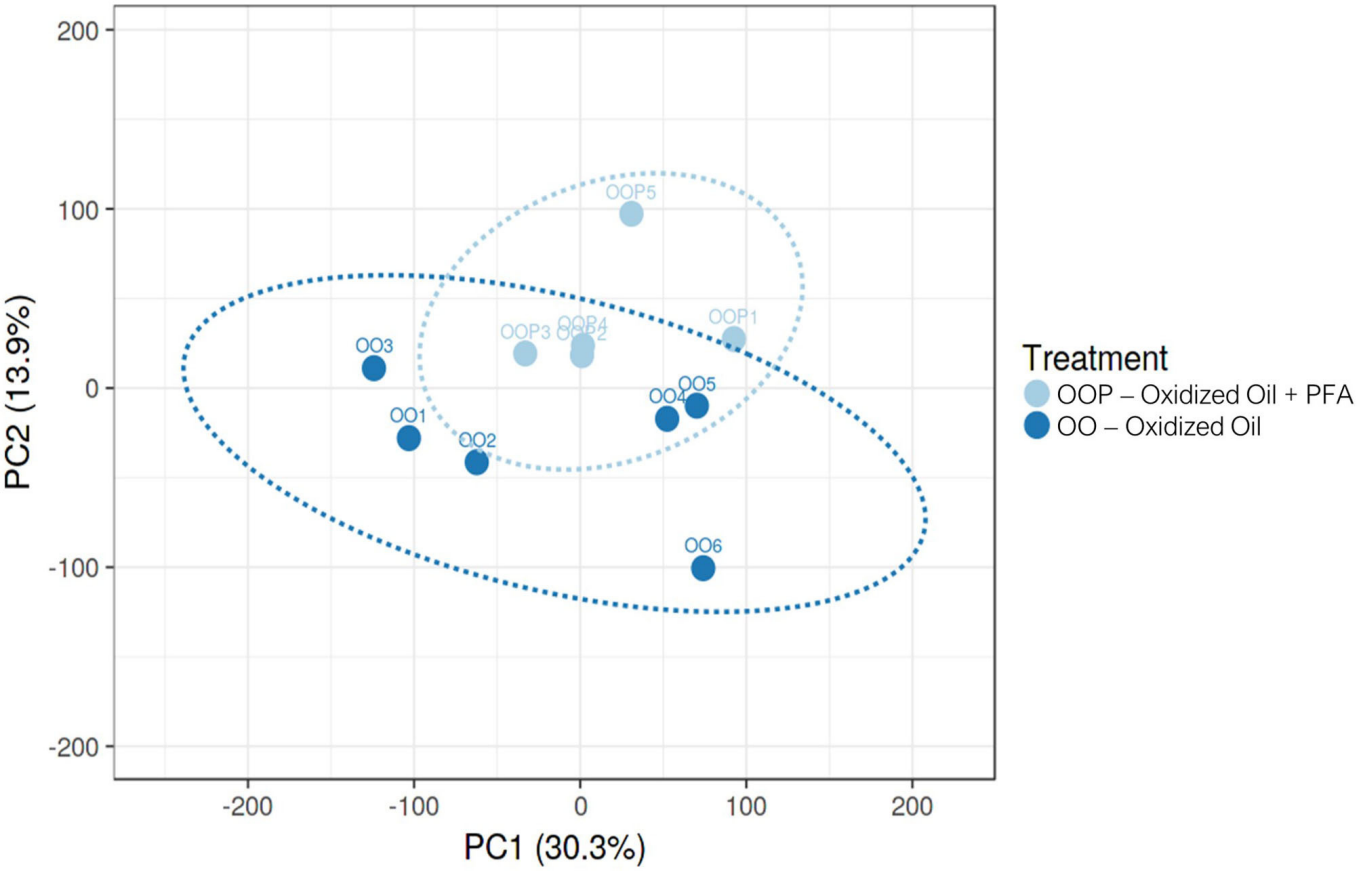
Principal component analysis on mRNA expression in the liver of piglets fed Oxidized Oil (OO) diet or Oxidized Oil + Phytogenic Feed Additive (OO + PFA). Analysis performed with Clustvis (prediction ellipses with 95% confidence interval). One piglet from the OO + PFA group was excluded from the RNA-Seq analysis due to technical issues observed during read quality control.

Considering all four groups were analyzed (unlike the ileum analysis), the LRT approach was preferred to the pairwise comparison and revealed significant changes in the expression of 2,762 genes across all experimental diets. From this list of genes, six gene clusters exhibiting similar gene expression pattern in each of the four groups were identified. Among them, the cluster 1 showed that the expression of 279 genes was strongly increased in the piglets fed OO, and partly reduced when fed OO + PFA (Figure 2A). The genes contained in this cluster are directly involved in the effect of the OO challenge as demonstrated by the overrepresentation analysis (Figure 2B). Indeed, many of those genes hit signaling pathways related to lipid oxidation, metabolism, and peroxisome. By contrast, very few pathways were significantly enriched in the five other clusters, indicating similar gene expression patterns but not associated with any biological functions (Supplementary Figure 1). This difference in the transcriptome profile between OO piglets and OO + PFA piglets can also be observed in Figure 3. Pairwise comparison was also performed between the control and PFA groups without OO, and four genes (*PLPPR2, ARHGEF28, RF00231*, and *DEF6*) were found differentially expressed with the cutoff values FDR ≤ 0.05 and fold change > |2|. Although enriched pathways were found in PFA when applying GSEA (e.g., HS-GAG degradation, resolution of sister chromatid cohesion, G1 phase, or RET signaling; FDR *p* < 0.05), the biological meaning was not very conclusive (Supplementary Table 2).

**Figure 4:**
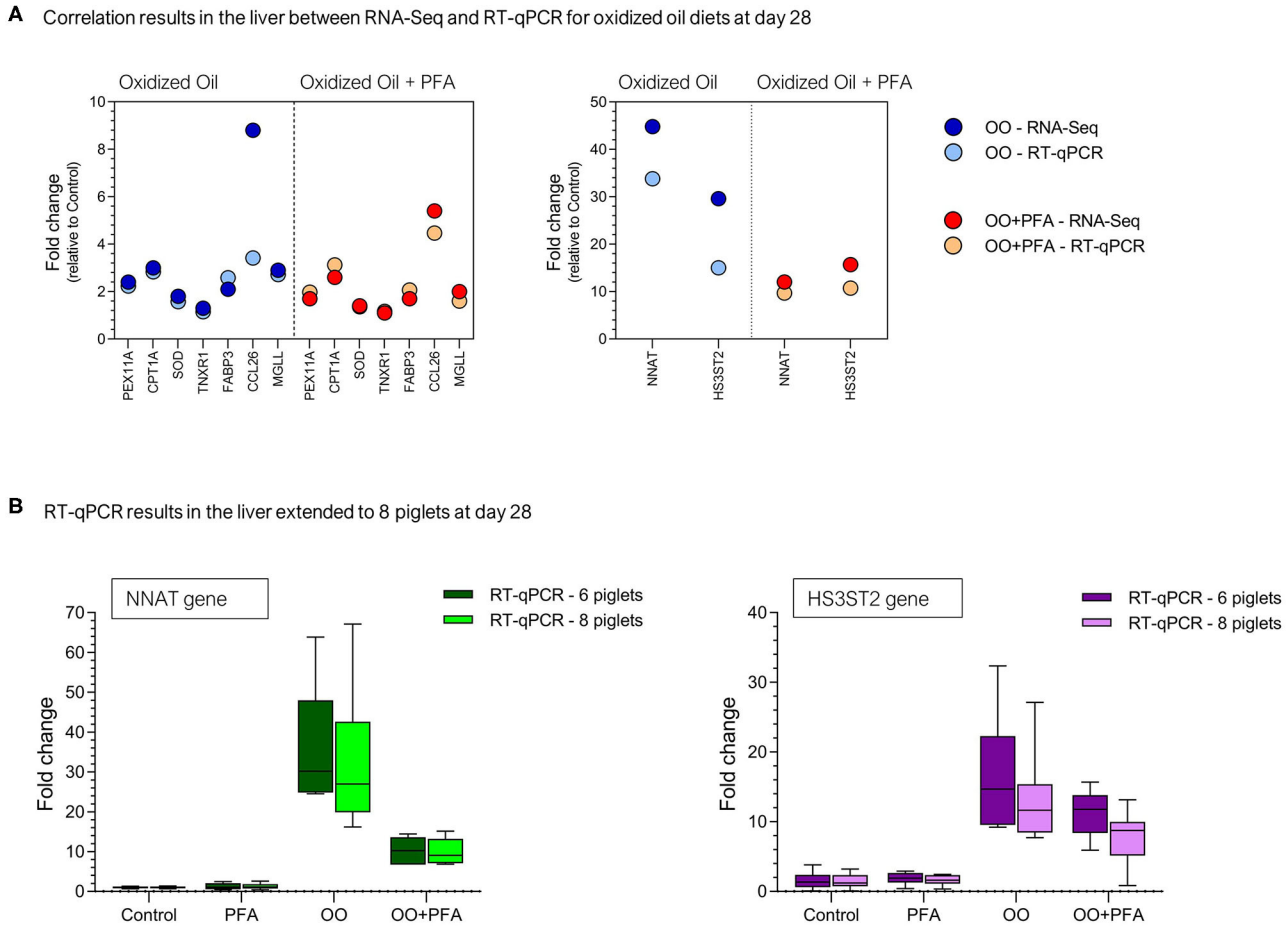
RT-qPCR validation on selected genes at day 28. **(A)** Correlation between the fold changes observed in RNA-Seq and RT-qPCR for the same piglets (*n* = 6). **(B)** Expression of the genes NNAT (Neuronatin) and HS3ST2 (Heparan sulfate glucosamine 3-O-sulfotransferase 2) via RT-qPCR in the liver of eight piglets (eight sampled initially but six used for RNA-Seq). PFA, Phytogenic Feed Additive; OO, Oxidized Oil; OO + PFA, Oxidized Oil + Phytogenic Feed Additive.

For confirmation of sequencing results, nine genes (*NNAT, HS3ST2, PEX11A, CPT1A, SOD1, TNXR1, FABP3, CCL26*, and *MGLL*) were quantified in the same liver samples by qPCR. The correlation analysis (18 X,Y pairs) between fold changes observed in RNA-Seq and in qPCR for OO or OO + PFA diets showed a Pearson correlation coefficient of *r* = 0.97 (*p* < 0.001). These results are presented in Figure 4A for the same liver samples (*n* = 6). Although the correlation is quite high, it seems that confirmation of the RNA-Seq results is very good for fold changes lower than 4, but partly in agreement for higher fold changes, especially in the OO diet (*CCL26* and *HS3ST2*). Given eight piglets were fed and sampled per diet in the experiment, the gene expression was extended to the piglets not selected for RNA-Seq analysis, namely, two more RNA samples per diet. The comparison between six or eight piglets on the expression of the most upregulated genes (*NNAT* and *HS3ST2*) is presented in Figure 4B.

## Discussion

In the present experiment, a standardized formulation of the PFA was used containing a combination of essential oils (i.e., oregano and caraway oils) and natural identical compounds (15). The main ingredients and active substances in PFA were carvacrol, linalool, methyl salicylate, thymol, and menthol. The beneficial effects of phytochemicals have been extensively demonstrated, especially essential oils used individually or as blends [review in (6, 13, 16)]. Although the formulations (plant extracts, essential oils, and single molecules) differ from each other in those studies, our results generated in the ileum of piglets fed PFA (in normal conditions) also revealed decreased activity of pathways and mediators associated with the innate immune response (IFN signaling, TNF signaling, and NF-κB activation). The anti-inflammatory effects of various essential oils and plant extracts have been mostly characterized *in vitro* in monocytes and activated macrophages, showing reduced activity of cytokines [reviewed in (17)]. In the duodenum and ileum of weaned piglets, polyphenol-rich plant products have been shown to reduce after 4 weeks the expression of pro-inflammatory cytokines (IL-1β, IL-8, TNF-α, and CCL2) (18). The concentration of two acute phase proteins was found significantly lower in the serum of weaned piglets fed a natural plant-derived supplement (alkaloid-based) compared to the control group (19). Essential oils, especially derived from oregano such as in the present study, have been shown to modulate both Nrf2 and NF-κB transcription factors to suppress intestinal inflammation (20–23). Reduced expression of the genes coding for NF-κB, TNF-α, IL-1β, and IL-6, as well as inactivation of the NF-κB p65 protein, was observed in the small intestine of pigs (21, 23). Curcumin (polyphenol) has been shown to block cytokine-mediated NF-κB activation and proinflammatory gene expression by inhibiting inhibitory factor IκB kinase (IKK complex) activity in intestinal epithelial cells (24). Similarly, anti-inflammatory actions of carvacrol seemed to be mediated through ERK1/2 and NF-kB in LPS-induced pro-inflammatory activation of macrophages (25). Our GSEA revealed that the ingredients of PFA reduced similarly the activity of genes associated with NF-κB signaling pathway, such as IKK-γ (IKBKG), IKK-β (IKBKB), IκB-α (NFKBIA), and NFKB2. The potential of phenolic compounds, such as carvacrol, to act on NF-κB was also demonstrated *in vitro* with caffeic acid phenethyl ester (found in a variety of plants), showing an inhibition of TNF-α-dependent NF-κB activation *via* direct inhibition of IKK (26). In the present study, the higher activity in the NF-κB signaling pathway in the control pigs compared to pigs fed PFA seems to be mediated *via* TNFR1 and TRAF6 (TNF Receptor Associated Factor 6). TRAF6 is also implicated in the regulation of type I IFN induction, and both TRAF6-mediated IRF7 activation and interferon alpha/beta signaling were found in GSEA. Several members of the interferon regulatory factors (IRFs) were found multiple times in these pathways, exhibiting lower activity in the PFA group. These proteins are crucial mediators in antiviral responses, but anti-inflammatory effects mediated by the IRF3 pathway (as well as NF-κB) have also been reported *in vitro* following the exposure of LPS-stimulated RAW cells to essential oil (27).

The lower abundance of innate immune cells in the gut could partly account for the reduced activity of pro-inflammatory mediators following ingestion of phytochemicals. The decreased number of intra-epithelial lymphocytes has already been reported in the small intestine of piglets fed similar compounds as in the PFA formulation, namely, carvacrol and thymol (28), and fed a plant extract containing carvacrol, cinnamaldehyde, and capsicum oleoresin (29). The use of RNA-Seq in the present trial allowed the unveiling of other small changes and pathways associated with innate immune response and stress, and not addressed with targeted approaches. Piglets fed with the phytogenic showed reduced expression of genes associated with some antimicrobial peptides and pathways belonging to extracellular matrix organization (collagen degradation and collagen chain trimerization). Interestingly, extracellular matrix remodeling is a common feature in inflammatory bowel disease due to tissue damage and destruction (30, 31). The expression of several of these genes (COL4A4, COL22A1, MMP8, COL4A3, COL17A1, COL11A2, COL9A1, COL26A1, MMP9, COL16A1, MMP15, COL8A2, MMP11, and COL18A1) was found increased in the control group, suggesting that PFA may reduce the basal activity of the extracellular matrix and maintain homeostasis in response to potential intestinal insult. Similar findings on matrix metalloproteinases and collagen chains were reported in the ileal mucosa of broiler chickens, indicating a protective effect of resin acids on intestinal barrier integrity (32). Overall, it seems that PFA is able to lower the basal level of certain components of the innate immune response, and this may potentially help the piglets to limit inflammation when facing stressors in pig production, such as demonstrated in weaned piglets fed plant extracts able to alleviate diarrhea caused by *Escherichia coli* (33).

In addition to their anti-inflammatory properties, phytogenic compounds are also known to protect against oxidative stress since phenolic OH groups (e.g., in thymol or carvacrol) can donate hydrogen atoms to free radicals and transform them into more stable products [reviewed in (11, 13, 16)]. Oxidative stress represents an important chemical mechanism that leads to biological damage, which, in turn, can affect growth performance, meat quality, and health in pigs, especially in modern high-performance production systems. In animals, malondialdehyde (MDA) is often used as a blood marker of oxidative stress since MDA is a terminal product of lipid peroxidation (caused by free radicals). In weaned pigs, neither a low-energy diet nor a blend of cinnamaldehyde and thymol affected the plasma concentration of MDA (34). However, dietary oregano oil (containing mainly carvacrol and thymol) was shown effective to alleviate the stress of transportation in finishing pigs such as seen with reduced MDA levels in the liver (35). Improvement of meat quality was reported in chickens fed either carvacrol or thymol *via* measurements of lipid oxidation, known as the major deterioration process for poultry meat (36).

The implementation of oxidative stress challenges allows a better evaluation of the antioxidant capacity of the phytochemicals tested. Among other techniques used for manipulating oxidative stress, adding oxidized lipids to the diet has been found very useful with several published trials, especially in fish and poultry [reviewed by Koch and Hill (37)]. As expected, the ingestion of moderately oxidized oil resulted in slow growth rate, but with improvement in the body weight gain of piglets challenged and supplemented with PFA (two-way ANOVA; *p* < 0.001 for oxidized oil challenge, *p* = 0.91 for diet, *p* = 0.033 for interaction; data not shown). The capacity of this experimental model to induce oxidative stress was confirmed based on our RNA-Seq findings. The challenge itself significantly affected the expression of 401 genes in the liver of pigs (pairwise comparison between both control diets, FDR ≤ 0.05 and fold change ≥ 2 or ≤ −2; data not shown). The highest fold increase recorded in our dataset was 44.8 for NNAT gene (neuronatin; validated by RT-qPCR), which is highly expressed during oxidative stress and inflammation in response to dietary excess such as obesity (38). The prediction pathway analysis clearly showed that the challenge affected genes related to fatty acid β-oxidation, peroxisome, or regulation of lipid metabolism by PPAR-α. Many genes from these pathways were enriched in the challenged group. Little is known about the global effect of such challenge on the transcriptome, but similar findings were also found in the liver of fish (39). Moreover, Ringseis and Eder (40) summarized the effects of oxidized fat on molecular regulators of lipid metabolism, and reported evidence for the activation of PPAR-α by oxidized fat. As indicated in their review, expression of PPAR-α itself remains largely unchanged in response to oxidized lipids, but they upregulate a comprehensive set of PPAR-α target genes. Among them, CPT1A and CPT2 (carnitine palmitoyltransferase) were also found upregulated (three-fold increase) in the present experiment. Activation of the Nrf2 pathway has also been reported in the study of Varady et al. (14), using similar oxidative stress model as in our study. However, the genes they found significantly increased in the pig livers (Nrf2-regulated genes) were not differentially expressed in our dataset. A closer look at the data revealed that the fold changes of those genes were very comparable to ours (from 1.4 to 1.8 increase). Standard threshold used in RNA-Seq (fold changes ≥ 2 or ≤ −2) as well as correction for multiple testing account for differences when comparing with literature.

The antioxidant properties of herbs and essential oils are reported to be effective in retarding or preventing the process of lipid peroxidation (16). Oral administration of carvacrol in rats for 21 days neutralized the hepatoxic effects induced by D-galactosamine on lipid peroxidative markers and liver tissue (41). The ability to prevent lipid peroxidation is associated with the scavenging properties of free radicals by phytochemicals (16, 42), and ingestion of oxidized fats results in generation of free radicals, such as reactive oxygen species (ROS) (14). Wei et al. (43) demonstrated in weaned piglets the ability of a blend of carvacrol and thymol to scavenge ROS during intestinal oxidative stress. The supplementation of PFA in the oxidized oil diet was partly able to neutralize the detrimental effects induced by the challenge on the liver transcriptome. Among the genes related to PPAR-α pathways (i.e., fatty acid β-oxidation, peroxisome; enriched in cluster 1, Figure 2), the effects were either no longer seen or reduced in average by 22% and up to 73% (fold change comparison between OO and OO+PFA diets). This latter finding was observed on the NNAT gene, previously discussed, with a fold increase of 12.0 in the liver of challenged pigs fed PFA instead of 44.8 without supplementation. We can hypothesize that the active components of PFA (i.e., carvacrol, linalool, and thymol) possess properties to partly remove exceeding free radicals coming from the oxidized oil and/or the oxidative stress, and thereby preventing further lipid peroxidation. In addition, this might help to decrease the workload of antioxidant enzymes and maintain their activities. Whether this scavenging mechanism of essential oils occurs in the intestine and/or the liver to limit oxidative stress is still unclear. It is known that these phenolic compounds, especially carvacrol and thymol, are almost completely absorbed in the proximal small intestine and the stomach of piglets [following single dose administration; (44)]. The metabolic fate of essential oil components depends on their individual chemical structure, but glucuronidation of thymol and carvacrol seems to be the major metabolic pathway in the liver, together with fast excretion in the urine (45). However, recovery of essential oils in the bile (carvacrol, thymol, linalool, and menthol) suggests enterohepatic circulation, increasing their occurrence in the gut and delaying their excretion (44, 45). Conjugated metabolites can be cleaved by enzymes in the intestinal microflora and also reabsorbed by epithelial cells of the intestine. Besides, the use of encapsulation such as in the present study increases the stability and bioavailability of those compounds, reducing the requirement for high dosage. The hepatoprotective effects of PFA against the oxidized lipids might have been the result of their interaction in both the liver and small intestine during the continuous feeding. Overall, this improvement on the liver health most likely contributed to the better growth observed in those pigs in comparison to the challenged pigs on basal feed. Although oxidative stress is common in animal production, such acute challenge is not to be expected under commercial conditions. Application of this model for evaluating antioxidant blend or vitamin E has been reported in literature (46–49), and these compounds were shown effective in ameliorating the negative effects of oxidized oil, but never fully, such as in our study.

The results of the present investigations showed that dietary supplementation of PFA modulated specific pathways related to NF-κB and the innate immune response in the ileum. Supplementation with PFA partly reduced the oxidative stress induced by the addition of the oxidized oil, but confirms that some ingredients from the formulation possess antioxidant properties. The use of RNA-Seq is still very limited in livestock species but allowed the generation of novel data on the molecular effects and host interaction with phytochemicals. This may contribute to a better understanding for the animal industry, with the ultimate goal of reducing antibiotic use.

## Materials and Methods

### Piglet Feeding Trial, Oxidized Oil Diet, and Tissue Collection

The animal experiment was approved by the office of the Lower Austrian Region Government, Group of Agriculture and Forestry, Department of Agricultural Law (approval code LF1-TVG-39/046-2017). The feeding trial was carried out at the Center for Animal Nutrition (Tulln, Austria). All related procedures were performed in compliance with the European Guidelines for the Care and Use of Animals for Research Purposes and the Austrian Law for Animal Experiments.

Weaned crossbred piglets (sow: Landrace × Large White, boar: Pietrain; 30 ± 2 days old) were obtained from a local producer. Thirty-two animals (16 males, 16 females) were selected based on equal body weight and housed on slatted floor pens under controlled environmental conditions. After an acclimatization period of 5 days, the piglets were allocated to one of four experimental diets (eight pigs/pen and one pen/diet). They received for 28 days either a basal diet (Control), a basal diet supplemented with Digestarom^®^, a PFA (150 g/t of feed; BIOMIN Holding GmbH, Austria), or the same two diets but with inclusion of oxidized oil at 10% (OO and OO + PFA). PFA is a combination of plant extracts (essential oils) and natural identical (synthetic) compounds. The main ingredients of PFA are oregano oil, caraway oil, carvacrol, methyl salicylate, and menthol. All the piglets had *ad libitum* access to the experimental feed and water. To evaluate the antioxidant capacity of PFA, addition of oxidized oil was used as a model of oxidative stress, and the same rapeseed oil was used as contained in the non-oxidized diets. The formulation for the normal diet and the oxidized oil diet is reported in Supplementary Table 3. This rapeseed oil was obtained locally (Süss Öl, Gemeinlebarn, Austria) and was not supplemented with any antioxidants. To prepare the oxidized oil, rapeseed oil was heated in a domestic fryer (2 × 10 L; PEF 10+10S, ASM Sautner GmbH, Austria) at a temperature of 185°C for 60 h, and peroxide values were determined according to ISO methods (3960:2017) (50) so that they reached ~50 mEq/kg of oil. The two oxidized oil diets contained 10% oxidized rapeseed oil for a predicted peroxide value in the final feed of 5 mEq/kg close to what has been reported in (14, 48, 49). The inclusion of the phytogenic compounds in the experimental diets was verified *via* the analysis of the concentration of the main active substances, and they were in the range of expected values (15).

During the experimental period, the health status of the piglets was checked daily. In addition, piglets were weighed weekly. On day 28, blood was collected from individual piglets by punctuation of the *Vena cava cranialis*. After centrifugation (1,500 × g, 20 min at 4°C), serum samples were stored at −80°C. Following blood sampling, piglets were anesthetized by intramuscular injection of ketamine (0.1 ml/kg b.w. Narketan^®^, Vétoquinol GmbH, Germany) and azaperone (0.03 ml/kg b.w. Stresnil^®^, Janssen-Cilag, Germany) and euthanized by intracardial injection of T61^®^ (0.1 ml/kg b.w., Intervet, Germany). The ileum was removed from all piglets, and the middle part was sampled and processed on ice. After cutting the ileum and rinsing with sterile saline solution (0.9%), small pieces were transferred into 1 ml of RNAlater (Ambion Inc., USA), stored overnight at 4°C, and transferred to −80°C until transcriptomics analysis. The same procedure was applied for the liver with cleaned small pieces transferred into 1 ml of RNAlater and stored the same way as for the ileum samples.

### RNA Extraction and RNA-Seq

Approximately 30 mg of ileal or liver tissues were disrupted *via* a bead-beating step, and the total RNA was extracted and purified with the RNeasy Mini Kit including DNase digestion on column (Qiagen, Germany). The concentration of isolated RNAs was estimated on a NanoDrop 2000 spectrophotometer (Thermo Scientific, USA) and RNA quality (RIN) with an Agilent 2100 Bioanalyzer (Agilent Technologies, Germany). The RIN was >9.0 for the RNA extracted from the ileum and >6.0 for the RNA extracted from the liver. The RNAs from five piglets per group (only unchallenged groups; two groups; 10 samples) were selected for the ileum and those from six piglets per group (unchallenged and challenged groups; four groups; 24 samples) were selected for the liver. Construction of RNA-Seq libraries was done with the Illumina stranded TruSeq RNA library including poly(A) enrichment. The final multiplexed libraries were sequenced on a NextSeq platform (Microsynth, Switzerland) generating ~60 Mio paired-end reads per sample (±20%; 30 Mio forward + 30 Mio reverse reads; 2^*^75 bp).

### RNA-Seq Analysis: Read Alignment and Counts, and Differential Expression Analysis

The RNA-Seq pipeline was implemented *via* the Snakemake workflow management system [version 5.2.4; (51)]. The configuration file contains the FASTQ file location, the experimental design, as well as global and step-specific parameters.

Sequencing quality was evaluated by FastQC software (http://www.bioinformatics.babraham.ac.uk/projects/fastqc/). Alignment was done with STAR aligner with the default parameters [version 2.6.1d; (52)] against the reference pig genome Sus_scrofa.Sscrofa11.1 (Supplementary Table 4). Read counts for each gene were estimated using HTSeq package [version 0.11.0; (53)] and aggregated to form a count table.

Differential expression was performed by using the R package DESeq2 [version 1.18.1; (54)], which uses a negative-binomial distribution to model the counts. Hypothesis testing was done with the Wald test for the ileum dataset (pairwise comparison) and with the Likelihood ratio test (LRT) for the liver dataset (full model to analyze all factors at once). Subsequently, raw *p*-values obtained either from the Wald test or the LRT were adjusted for multiple comparison testing using the Benjamini–Hochberg procedure (55), and genes were considered differentially expressed when adjusted *p*-values ≤ 0.05.

### Functional Enrichment Analysis: Overrepresentation Analysis and GSEA

Two different approaches were used according to our datasets, namely, ileum and liver datasets.

GSEA was performed on the ileum dataset with the GSEA software [version 4.0.3; (56)]. GSEA is the second generation of non-topology-based approaches (57) and eliminates the dependency on the gene selection criteria by taking all gene expressions into consideration. It assumes that phenotypic differences are manifested by small but consistent changes in a set of genes (such as expected with the ileum dataset, no challenge). Normalized counts from DESeq2 were used as input data in the software, and GSEA was run with the following parameters: c2.cp.reactome.v7.0.symbols.gmt (Reactome gene sets, 1,554 gene sets; curated gene sets in the MSigDB collections), 10 (number of permutations), no_collapse (gene symbols), and gene_set (permutation type; recommended for less than seven samples per phenotype/group). Gene sets were considered significantly enriched with FDR (*q*-values) ≤ 0.05 and nominal *p*-values ≤ 0.05 of the normalized enrichment scores.

Regarding the liver dataset, in order to identify expression pattern across conditions, the degPattern clustering function from the R package DEGreport (version 1.14.1) was applied to the VST transformed count matrix containing the genes with an adjusted *p*-value ≤ 0.1 (issued from LRT approach in DESeq2). Then, for each cluster of genes, a functional enrichment of Sus_scrofa.Sscrofa11.1 GO terms was performed using the enricher function of the R package clusterProfiler [version 3.6.0; (58)]. The conversion table Ensembl gene ID-GO term name was retrieved from the R package BioMart (59). In addition, exploratory analysis was performed with Clustvis (60).

### qPCR Validation of RNA-Seq Results

For verification of RNA-Seq results, we conducted quantitative reverse transcription PCR (RT-qPCR) assays on the same RNA samples as used for RNA-Seq (RNA isolation already described above). In addition, we extended this verification to all the piglets that were sampled at the same time as the piglets used for RNA-Seq (RT-qPCR on a total of 8 pigs/group).

The total RNA was used for first-strand cDNA synthesis using Maxima H Minus First Strand cDNA synthesis kit (Thermo Fisher Scientific) according to standard procedures. All the primers (Supplementary Table 5) were designed with the software Primer3 [(61); https://primer3plus.com/primer3web/primer3web_input.htm] and pre-experimentally validated (qPCR tests including program of temperature gradient and followed by electrophoresis gel for amplicon size confirmation). All RT-qPCR reactions were conducted on the Mastercycler ep Realplex (Eppendorf) using SYBR green chemistry (Kapa SYBR Fast Universal, Sigma). The thermal cycle conditions were as follows: 1 cycle of pre-incubation at 95°C for 3 min, 40 cycles of amplification (95°C for 10 s, 60°C for 20 s, and 72°C for 20 s), and melting curve program included at the end of the run. Relative gene expression was calculated using the 2^−ΔΔCt^ method with the geometric mean of the Cts from the four housekeeping genes HPRT1, RPL32, GAPDH, and ACTB serving for normalization [subtracting the Ct-values of individual target genes from the Ct-value of the housekeeping genes of the same sample (ΔCt-values)]. Next, the mean ΔCt for each experimental group and target gene was calculated and subsequently used for statistical evaluation and expressing the fold change (=2^−ΔΔCt^ value).

### Statistics Other Than for RNA-Seq Analysis

Statistical analysis was performed using GraphPad Prism version 8 for Windows (GraphPad Software, La Jolla, CA, USA). The Shapiro–Wilk method was used to test the normality of the data distribution. Factorial or two-way ANOVA (source of variation: OO, PFA, and interaction) followed by Tukey's multiple comparisons test was used for the body weight gain. The Pearson correlation coefficient was also determined as a measure of the linear correlation between qPCR fold changes and NGS fold changes.

## Data Availability Statement

The datasets presented in this study can be found in online repositories. The names of the repository/repositories and accession number(s) can be found below: https://www.ncbi.nlm.nih.gov/geo/, GSE159583.

## Ethics Statement

The animal study was reviewed and approved by Office of the Lower Austrian Region Government, Group of Agriculture and Forestry, Department of Agricultural Law.

## Author Contributions

BG and GS designed the experiment and conceived the whole research study. BG and SF-S supervised the feeding trial and the samplings. BG and SI extracted the RNA from the tissue samples and coordinated the RNA-Seq. JLC executed the bioinformatics analysis. SF-S provided support in the bioinformatics activities. BG and JLC performed the functional enrichment analysis and prepared the manuscript. SI carried out the RT-qPCR analysis. BG, JLC, and SI analyzed and interpreted the data. All authors read and approved the manuscript.

## Conflict of Interest

All authors were employed by BIOMIN Holding GmbH.

## Publisher's Note

All claims expressed in this article are solely those of the authors and do not necessarily represent those of their affiliated organizations, or those of the publisher, the editors and the reviewers. Any product that may be evaluated in this article, or claim that may be made by its manufacturer, is not guaranteed or endorsed by the publisher.
