# Supplementary figures and images for: Exploring With Transcriptomic Approaches the Underlying Mechanisms of an Essential Oil-Based Phytogenic in the Small Intestine and Liver of Pigs
Source: Front Vet Sci. 2021 Aug 11;8:650732. doi: 10.3389/fvets.2021.650732 (PMC8386756; doi:10.3389/fvets.2021.650732)

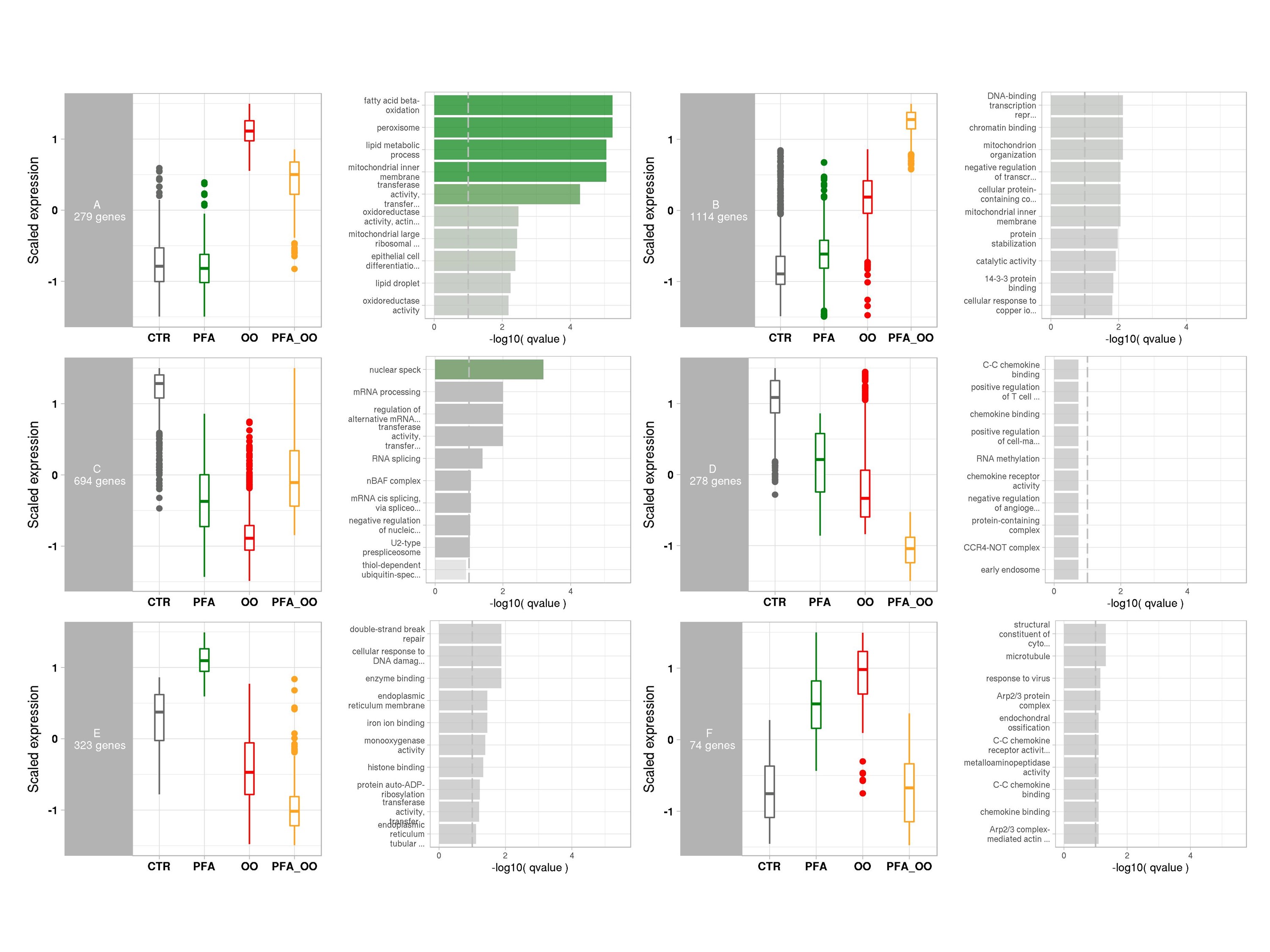

Supplement: Supplementary file 1 [file Image_1.jpg]
