# Supplementary material for: Exploring With Transcriptomic Approaches the Underlying Mechanisms of an Essential Oil-Based Phytogenic in the Small Intestine and Liver of Pigs
Source: Front Vet Sci. 2021 Aug 11;8:650732. doi: 10.3389/fvets.2021.650732 (PMC8386756; doi:10.3389/fvets.2021.650732)
Supplement: Supplementary file 4 [file Table_3.docx]

**Supplementary Table 3:** composition of the diets

| Item | Normal diet | Oxidized oil diet |
| --- | --- | --- |
|  | | |
| Ingredient (% of the diet) |  |  |
| Wheat | 10.00 | 9.00 |
| Barley | 29.70 | 26.73 |
| Corn | 10.08 | 9.07 |
| Fresh rapeseed oil | 0.45 | 0.41 |
| Oxidized rapeseed oil | - | 10.00 |
| Fullfat soya | 15.48 | 13.93 |
| Maize pressure cooked | 10.08 | 9.07 |
| Potatoe protein | 5.04 | 4.54 |
| Dextrose | 5.04 | 4.54 |
| Palm kernel, cocos fat | 3.78 | 3.40 |
| Lactose | 3.78 | 3.40 |
| Lignocellulose | 1.35 | 1.22 |
| Mono calcium phosphate | 1.22 | 1.10 |
| Calcium carbonate | 0.95 | 0.86 |
| Sodium chloride | 0.45 | 0.41 |
| Magnesium phosphate | 0.27 | 0.24 |
| Vitamin and mineral premix^1^ | 0.89 | 0.80 |
| L-Lysine | 0.70 | 0.63 |
| L-Threonine | 0.26 | 0.23 |
| DL-Methionine | 0.27 | 0.24 |
| L-Valine | 0.14 | 0.13 |
| L-Tryptophane | 0.06 | 0.05 |
| Sweetener | 0.02 | 0.02 |
|  | | |
| ME, kcal/kg (calculated) | 3272 | 3641 |

^1^The vitamin and mineral premix provided the following per kg diet: 6.1 g calcium; 6.1 g phosphorus; 2.1 g sodium; 1.7 g magnesium; 14.0 g lysin; 5.3 g methionine; 9.1 g threonine; 2.5 g tryptophan; 9.4 g valin; 15600 IU vitamin A; 2000 IU vitamin D3; 150 mg vitamin E; 150 mg vitamin C; 4.0 mg vitamin K3; 3.1 mg vitamin B1; 8.5 mg vitamin B2; 5.8 mg vitamin B6; 50 µg vitamin B12; 62 mg nicotinic acid; 22 mg pantothenic acid; 900 mg choline chloride; 1250 µg folic acid; 150 µg biotin; 25 mg copper; 120 mg zinc; 60 mg manganese; 1.2 mg iodine; 0.5 mg selenium.
