# Supplementary material for: Exploring With Transcriptomic Approaches the Underlying Mechanisms of an Essential Oil-Based Phytogenic in the Small Intestine and Liver of Pigs
Source: Front Vet Sci. 2021 Aug 11;8:650732. doi: 10.3389/fvets.2021.650732 (PMC8386756; doi:10.3389/fvets.2021.650732)
Supplement: Supplementary file 6 [file Table_5.docx]

**Supplementary Table 5:** Nucleotide sequence of primers for real-time qPCR

| **GENE** | **PRIMER SEQUENCE** | **AMPLICON SIZE** | **ENSEMBL ACCESS #** |
| --- | --- | --- | --- |
| *Housekeeping genes* | | | |
| ***HPRT1*** | F (300 nM) CAAGTTTGTGGTAGGCTATGC  R (300 nM) TGGCCACAGAACTAGAACATGT | 191 bp | ENSSSCG00000034896 |
| ***RPL32*** | F (300 nM) GGAGAAGGTTCAAGGGCCAG  R (300 nM) GGCTTTGCGGTTCTTGGAAG | 196 bp | ENSSSCG00000035811 |
| ***GAPDH*** | F (300 nM) TTGCCCTCAACGACCACTTC  R (300 nM) GTCCAGGGGCTCTTACTCCT | 121 bp | ENSSSCG00000000694 |
| ***ACTB*** | F (300 nM) GGACTTCGAGCAGGAGATGG  R (300 nM) AGGTAGTTTCGTGGATGCCG | 175 bp | ENSSSCG00000007585 |
| *Target genes for qPCR validation of RNA-Seq results* | | | |
| ***CCL20*** | F (300 nM) AACATCACAGCACTCCCAGG  R (300 nM) ATCGGAGGCAGCAGTCAAAG | 175 bp | ENSSSCG00000016254 |
| ***IRF7*** | F (300 nM) GGACCCCACTGACCCTCATA  R (300 nM) GCCTCAGCCTCTCACCAGTA | 167 bp | ENSSSCG00000012853 |
| ***TRAF2*** | F (300 nM) TTCTACACGAGCAGGTACGG  R (300 nM) TTCTGGTCCAGCAGCATCAG | 175 bp | ENSSSCG00000005838 |
| ***TOLLIP*** | F (300 nM) GCAACAGCAACAGCAGATCC  R (300 nM) GGTCACGCCGTAGTTCTTCG | 135 bp | ENSSSCG00000033033 |
| ***GSDMD*** | F (300 nM) GACGCCATGCACCTTGAAAG  R (300 nM) CACCTCCTTCTGCGTCTGG | 122 bp | ENSSSCG00000006955 |
| ***PEX11A*** | F (300 nM) CTTCACCAACCAGACCCAAG  R (300 nM) ACTGGCCTCCAGTTTCTTGA | 138 bp | ENSSSCG00000027052 |
| ***CPT1A*** | F (300 nM) AGGCCCTCAGGCAGATCTAT  R (300 nM) GGTCGATCTTGGCGTACATT | 161 bp | ENSSSCG00000012880 |
| ***NNAT*** | F (300 nM) ACTCGCTCTCAACCACCAAC  R (300 nM) CTGTGTCCCTGGAGGATTTC | 174 bp | ENSSSCG00000007336 |
| ***HS3ST2*** | F (300 nM) CTCTCCGGTGTCAACCACTC  R (300 nM) GGCCTCTTGAGTGACGAAAT | 272 bp | ENSSSCG00000036475 |
| ***SOD1*** | F (300 nM) GTGCAGGGCACCATCTACTT  R (300 nM) TCACCTTCAGCCAGTCCTTT | 85 bp | ENSSSCG00000021355 |
| ***TNXR1*** | F (300 nM) GGAGCTTCCCGAGATGTATG  R (300 nM) TGCAACCTACATTCACACACG | 179 bp | ENSSSCG00000000843 |
| ***CCL26*** | F (300 nM) CTGCTTCCAATACAGCCACA  R (300 nM) ATTTTTGCACCCATGTTTCC | 154 bp | ENSSSCG00000039874 |
| ***FABP3*** | F (300 nM) GCACCTGGAAGCTAGTGGAC  R (300 nM) AAACTCCACTCCCAGCTTGA | 196 bp | ENSSSCG00000036883 |
| ***MGLL*** | F (300 nM) GAGCCAGGACAAGACACTGA  R (300 nM) TCCTTTGGGAGACCCATGTA | 118 bp | ENSSSCG00000024134 |

All primers were used at a final concentration of 300 nM. DNase treatment was applied on all samples, and genomic DNA contamination was verified in the qPCR assays with the inclusion of RNA samples treated with DNase and non-reverse transcribed (ΔCt > 8 between cDNA and RNA treated with DNase and non-reverse transcribed samples was considered non-significant).

CCL20, C-C motif chemokine ligand 20; IRF7, interferon regulatory factor 7; TRAF2, TNF receptor associated factor 2; TOLLIP, toll interacting protein; GSDMD, gasdermin D;; PEX11A, Peroxisomal biogenesis factor 11 alpha; CPT1A, Carnitine palmitoyltransferase 1A; NNAT, Neuronatin; HS3ST2, Heparan sulfate-glucosamine 3-sulfotransferase 2; SOD1, Superoxide dismutase 1; TNXR1, Thioredoxin reductase 1; CCL26, C-C motif chemokine ligand 26; FABP3, Fatty acid binding protein 3; MGLL; Monoglyceride lipase.
